# Improved systemic and mucosal antibody responses with a CCR10 ligand adjuvant

**DOI:** 10.1186/1742-4690-9-S2-P203

**Published:** 2012-09-13

**Authors:** N Hutnick, DJ Myles, A Ginsberg, AS Khan, J Yan, Z Moldoveanu, J Mestecky, PA Marx, M Kutzler, DB Weiner

**Affiliations:** 1 University of Pennsylvania, Philadelphia, PA, USA; 2 Inovio Pharmaceuticals, Blue Bell, PA, USA; 3 University of Alabama, Birminham, AL, USA; 4 Tulane University, Covington, LA, USA; 5 Drexel University, Philadelphia, PA, USA

## Background

The induction of potent mucosal immune responses will be critical for an effective HIV vaccine, However, a major limitation of current vaccine development is the ability to induce mucosal antibodies by a systemic, non-replicating vector. To address this inadequacy, we have hypothesized that encoding instructions for immune cell targeting to the mucosa in the form of MEC, a mucosal chemokine adjuvant delivered as a plasmid can redirect immune responses in vivo. MEC (CCL28) is normally expressed by epithelium in the skin, lungs, and intestines and it functions to attract CCR10 expressing plasmablasts locally.

## Methods

IIndian rhesus macaques were vaccinated using EP delivery with either a pcon SIVmac239 gag, pol, SIVsm unmatched E660 env vaccine delivered IM alone (n=5), with CCL28 (MEC, n=5) or a plasmid expressed H1 HA Influenza vaccine alone (n=4) or with MEC (n=4). SIV Vaccinated animals and 6 naïve controls were challenged vaginally twice weekly for four weeks with 500TCID50 SIVsmE660.

## Results

The inclusion of a CCR10 ligand adjuvant enhanced vaginal and serum IgG and IgA titers compared with DNA alone. In Flu vaccinated animals functional HAI antibody titers were significantly elevated and above the 1:40 titer required for protection in humans with just a single dose of H1HA delivered with the MEC adjuvant. Following SIV challenge monkeys vaccinated with a CCR10 adjuvant showed 89% protection from the establishment of infection compared 40% with DNA alone with only 16% of the naïve animals.

## Conclusion

Mucosal and systemic antibody responses were enhanced with the inclusion of a CCR10 ligand adjuvant. Dose sparing was also observed. DNA vaccination alone improved challenge outcome, and this was further enhanced by the inclusion of a CCR10 ligand adjuvant. The inclusion of mucosal homing chemokines represents a novel approach to induce improved mucosal immune responses by non-live systemic immunization of relevance to HIV infection.

